# Health system strengthening: a qualitative evaluation of implementation experience and lessons learned across five African countries

**DOI:** 10.1186/s12913-017-2662-9

**Published:** 2017-12-21

**Authors:** Felix Cyamatare Rwabukwisi, Ayaga A. Bawah, Sarah Gimbel, James F. Phillips, Wilbroad Mutale, Peter Drobac, the AHI PHIT Partnership Collaborative, Ahmed Hingora, Dominic Mboya, Amon Exavery, Kassimu Tani, Fatuma Manzi, Senga Pemba, James Phillips, Almamy Malick Kante, Kate Ramsey, Colin Baynes, John Koku Awoonor-Williams, Ayaga Bawah, Belinda Afriyie Nimako, Nicholas Kanlisi, Elizabeth F. Jackson, Mallory C. Sheff, Pearl Kyei, Patrick O. Asuming, Adriana Biney, Roma Chilengi, Helen Ayles, Moses Mwanza, Cindy Chirwa, Jeffrey Stringer, Mary Mulenga, Dennis Musatwe, Masoso Chisala, Michael Lemba, Wilbroad Mutale, Peter Drobac, Felix Cyamatare Rwabukwisi, Lisa R. Hirschhorn, Agnes Binagwaho, Neil Gupta, Fulgence Nkikabahizi, Anatole Manzi, Jeanine Condo, Didi Bertrand Farmer, Bethany Hedt-Gauthier, Kenneth Sherr, Fatima Cuembelo, Catherine Michel, Sarah Gimbel, Bradley Wagenaar, Catherine Henley, Marina Kariaganis, João Luis Manuel, Manuel Napua, Alusio Pio

**Affiliations:** 1 Partners in Health-Inshuti Mu Buzima, Kigali, Rwanda; 2 Heilbrunn Department of Population and Family Health, Columbia University Mailman School of Public Health, New York City, NY USA; 3 Department of Global Health, University of Washington, Seattle, WA USA; 4 University of Zambia School of Medicine, Lusaka, Zambia; 5 University of Global Health Equity, Kigali, Rwanda; 6 Department of Global Health and Social Medicine, Harvard Medical School, Boston, MA USA; 7 Division of Global Health Equity, Brigham and Women’s Hospital, Boston, MA USA

**Keywords:** Africa, Global health, Health system strengthening, Implementation, Research

## Abstract

**Background:**

Achieving the United Nations Sustainable Development Goals in sub-Saharan Africa will require substantial improvements in the coverage and performance of primary health care delivery systems. Projects supported by the Doris Duke Charitable Foundation’s (DDCF) African Health Initiative (AHI) created public-private-academic and community partnerships in five African countries to implement and evaluate district-level health system strengthening interventions. In this study, we captured common implementation experiences and lessons learned to understand core elements of successful health systems interventions.

**Methods:**

We used qualitative data from key informant interviews and annual progress reports from the five Population Health Implementation and Training (PHIT) partnership projects funded through AHI in Ghana, Mozambique, Rwanda, Tanzania, and Zambia.

**Results:**

Four major overarching lessons were highlighted. First, variety and inclusiveness of concerned key players (public, academic and private) are necessary to address complex health system issues at all levels. Second, a learning culture that promotes evidence creation and ability to efficiently adapt were key in order to meet changing contextual needs. Third, inclusion of strong implementation science tools and strategies allowed informed and measured learning processes and efficient dissemination of best practices. Fourth, five to seven years was the minimum time frame necessary to effectively implement complex health system strengthening interventions and generate the evidence base needed to advocate for sustainable change for the PHIT partnership projects.

**Conclusion:**

The AHI experience has raised remaining, if not overlooked, challenges and potential solutions to address complex health systems strengthening intervention designs and implementation issues, while aiming to measurably accomplish sustainable positive change in dynamic, learning, and varied contexts.

## Background

In 2000, the United Nations Millennium Declaration was signed by 189 member countries. Following the adoption of the Declaration, a set of eight Millennium Development Goals (MDGs) was established as a target for global development and poverty eradication efforts [1, 2]. The drive to produce results for the health-related MDGs, including reducing maternal and child mortality and stemming the HIV epidemic, led many stakeholders to focus on disease-specific programs with variable results [3, 4]. There has been increasing recognition that disease-specific interventions will be more effective and sustainable when linked to improvements in the broader health system, including a strong focus on primary health care [5, 6]. Reflecting these lessons learned, broader health systems strengthening and primary health care are now seen as core to achieving Universal Health Coverage (UHC) and critical to meeting the new health-related United Nations Sustainable Development Goals (SDGs) [7, 8].

In 2009, the World Health Organization (WHO) identified six key components (building blocks) that need to be addressed to effectively strengthen the systems and inputs required for effective health care delivery [1]. However, the design and implementation of health systems strengthening (HSS) interventions is inherently complex and requires an understanding of the dynamic interplay between these components, institutions, communities and individuals, as well as adaptation to the initial and changing local and national context [9, 10]. Extracting the key learnings from this work is important to be able to inform current and future efforts to more effectively strengthen the care delivery and supporting systems needed to improve population health. Reflecting this complexity, there has been a growing use of a systems thinking approach in the design, implementation and evaluation of complex, multiple-level interventions, including HSS interventions in sub-Saharan Africa. In addition, the emergence of implementation science as a discipline has also resulted in new frameworks and models to inform decision-makers on how to best design, implement, and adapt interventions to the changing environments in which they are working and produce new knowledge for more effective strategies and implementation [11, 12].

Since 2009, the African Health Initiative (AHI) Population Health Implementation and Training (PHIT) partnership projects, funded by the Doris Duke Charitable Foundation (DDCF), has supported individual health system strengthening projects in five sub-Saharan African countries: Ghana, Mozambique, Rwanda, Tanzania, and Zambia [5]. The overarching framework for design and reporting of the interventions was the WHO Health Systems Building Blocks, which was one of the dominant HSS models at the time of the onset of the initiative [1]. PHIT partnership projects within each country built on existing relationships and included representatives from African and U.S. universities, implementing non-governmental organizations (NGOs), and the country’s Ministry of Health (MOH). These partnerships were tasked with designing, implementing, and evaluating large-scale primary health care delivery and workforce training interventions over a five to seven-year period. Although each partnership employed different HSS strategies appropriate to their respective context, all were designed to improve a common set of population health outcomes [5].

While detailed design and outcomes of the projects will be described elsewhere, our goal in this qualitative analysis was to explore the main shared intervention components and contextual factors that influenced the design and implementation process, and ultimately success, from the implementation leaders’ perspective. This information will help to explain and highlight common experiences and lessons learned from HSS intervention implementation across the PHIT sites; and contribute to efforts to better understand core elements needed to more rapidly spread interventions designed to strengthen primary health care delivery and efforts towards quality Universal Health Coverage.

## Methods

This study is a retrospective qualitative evaluation of the implementation experience and lessons learned from the district- and provincial-level health systems strengthening interventions in the five PHIT partnership projects in Ghana, Tanzania, Rwanda, Mozambique, and Zambia (Table 1). Each of the five PHIT projects implemented interventions in one or more districts, with catchment areas ranging from 250,000 to 1.5 million people (Table 1) and are described in detail in earlier papers [5, 6, 13–16].

**Table 1:** PHIT project summaries

Country	Partners involved	Catchment area	Health system strengthening intervention components *(Informed by the six WHO building blocks of a health system framework)*	Recommendable innovative programs/models/component
Ghana (Awoonor et al. 2013) [13]	Ghana Health Service Policy, Planning, Monitoring and Evaluation Division Navrongo Health Research Centre University of Ghana School of Public Health Columbia University Mailman School of Public Health	500,000 people (District health system in Upper East Region, Ghana)	Extended Newborn Service; IMCI; use of community health; data utilization; strengthening project leadership at all levels of the District health system	Community Health Nurse program Improvement of transport of obstetric emergencies from the community health posts to higher level facilities able to provide expanded emergency obstetric care
Mozambique (Sherr et al. 2013) [3]	University of Washington Health Alliance International University of Eduardo Mondlane Mozambique Ministry of Health	1,500,000 people (13 districts in Sofala Province)	Strengthening district health management systems and improving delivery of integrated primary health care	Beira Operations Research Center district−/facility-level data quality assessments Data utilization tools for district-level performance review process
Rwanda (Drobac et al. 2013) [6]	University of Rwanda College of Medicine and Health Sciences Rwanda Ministry of Health Harvard Medical School Brigham and Women Hospital Partners In Health	560,000 people: one and one-half rural districts	Targeted support for health facilities, quality improvement initiatives, strengthened the network of community health workers and improved monitoring and evaluation	Clinical Mentorship and Quality Improvement (MESH-QI model) Integrated mentoring and QI Collaborative to reduce Neonatal Mortality Operational/Implementation research capacity/skills building program
Tanzania (Ramsey et al. 2013) [15]	Ifakara Health Institute Columbia University Mailman School of Public Health Tanzania Training Center for International Health Council Health Management Teams	857,000 people (Kilombero, Ulanga and Ufiji Districts, Murogoro Region, Tanzania)	Introduction of a new cadre of Community Health Agents (CHAs) into a general program of health systems strengthening and referral. Supervisory systems to support the CHA. District-wide emergency referral strengthening intervention	Community Health Agent program
Zambia (Stringer et al. 2013) [16]	Zambart Centre for Infectious Disease Research in Zambia	559,000 people (Lusaka Province)	Clinical protocols for health care quality improvement. Community health workers to actively improve the referral system	Clinic supporters as trained CHWs Standardized Protocols and forms for patient screening Forms for patients consultations

### Data collection

Data were gathered through key informant interviews of the implementation leaders from each of the five PHIT projects present at a cross-site meeting of the AHI held in Rwanda in October 2015. In addition, we conducted a rapid desk review of available program documents, including publications from the country projects, and annual and six-month reports submitted to DDCF.

**Fig. 1:**
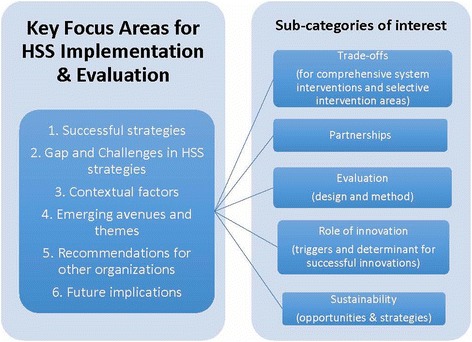
Key Focus Areas for HSS Implementation & Evaluation

Six semi-structured interviews were conducted, including at least one participant from each country team. Some of the candidates for the interviews represented two country projects (Ghana and Tanzania), as these projects had been linked and the informants had been involved with both interventions. All of the key informants had been engaged in the project from the conception phase, grant development, intervention design and implementation through to the impact evaluation phase. All interviews were conducted in English, recorded and transcribed. The interviews were conducted by a qualitative research expert who was not involved in the implementation of any of the projects. The interviews were structured in four segments corresponding to phases of the implementation experience: 1) intervention design and initial implementation, including contextual factors influencing decisions; 2) evolution of intervention over time, including contextual factors influencing adaptations; 3) learning across PHIT sites, and; 4) lessons learned for replication (see Fig. 1).

### Data analysis

Information obtained during the interviews was organized according to six domains of the WHO health system building blocks framework and adapted for the purposes of this study to understand the steps of each project [1]. Each of the key domains was further broken down into sub-categories to understand details of the teams’ interventions and implementation experiences. The interviews and progress reports were coded using an a priori approach and were used to complement missing data points from the interviews. The codebook was designed to capture themes related to the implementation and evaluation of the teams’ HSS interventions.

Inter-coder agreement was assessed after the primary round of coding to ensure no changes to the codebook were needed. Key findings were compiled by all coders and developed into a formal report as the deliverable. All coding was completed using Dedoose ver 6.2.10.

A secondary thematic analysis was conducted using the Consolidated Framework for Implementation Research (CFIR) to explore the contextual factors that influenced implementation success and need for adaptation (Table 2). The information gathered from each PHIT project was analyzed using the CFIR domains to identify core contextual factors influencing the intervention’s implementation success or failure [17]. CFIR was chosen for its recognition by recent implementation researchers as an important lens to understand the design, implementation, challenges, and successes of HSS interventions [18, 19]. The framework provided us a rich and validated taxonomy that enabled an in-depth exploration of the implementation of HSS interventions across the diverse contexts and communities [20, 21]. We carried out a thematic analysis of the interviews using the five CFIR domains: 1) intervention characteristics; 2) outer setting; 3) inner setting (the implementing organizations); 4) individual characteristics, and; 5) the implementation process. The full use of the CFIR model (i.e. scoring of constructs) was beyond the scope of this analysis, as the intention was not to compare the implementation across the sites and our sample size was small.

**Table 2:** Selected Contextual Factors influencing implementation success

CFIR domains	Facilitators	Key informants’ quotes	Barriers	Key informants’ quotes
Intervention characteristics	Embeddedness in the public system and close integration into national strategies;	*“Because it is an opportunity that we have been looking for a long time. We had a program that went through a rigorous research as far back as 1994 that proved the intervention that if you put nurses in the community and work closely with the community members and putting all the structures and equipment, everything, you will improve health outcomes.”*	Difficult to adapt to context due to evaluation framework (RCT or other fixed designs)	*“Because to actually work through the health system would’ve prevented us from being able to conduct an experiment. That tension was salient throughout the project, even from when we were working with the local government authorities because it was like why are you setting it up this way?”* *“The spirit of PHIT is very much one of implementation. Our project is a research project and I sense that that has put us always at a disadvantage compared to other PHIT projects which were directly go in and improve this.”*
	Innovation	*“…that was the situation that we had, this project came as a first project that was looking the entire health system, how do we make the system stronger …this is a project that will sit…right in the middle of the health system.”*		
	Built on existing partnerships	*“I think you have to subordinate the global expertise and technocratic input to context about what might work in that setting because to influence a system, you have to have people on board and you have to adapt their perspectives and their perceptions.”*		
	Plan to utilize data and adaptability	*“We were in equipoise (questionable vocabulary) [sic] and as researchers the best thing is to put the concept to the test, get results, and then see if it works, start now working towards implementation. I think that it is reasonable to say that it was our scientific intent to generate evidence before we recommend.”*		
	Rigorous evaluation framework	*“…based on our understanding of the need to have evidence- based recommendations and adjustments to the system; we didn’t want to say, 'We have this great quality improvement idea, let’s implement!' No, we said, 'We have these great ideas. Do they really work? We don’t know. Can they really contribute to improved quality improvement and improved mortality eventually?'”*		
Outer setting	Local individual expertise	*“The second thing was that we always concentrated on putting nurses in the community. We never talked about midwives*…*The midwife focuses also to address the inherent cultural practices that every community, every home, every clan in the district, in the community has a midwife.”*	Intermittent change in national policies priorities.	*“I think one of the challenges of working with the government is they have a lot of priorities and sometimes the national will say on a Monday, “You need to do this by Wednesday!” So all their planning kind of goes out the door. So understanding that they have pressures on their time so that they couldn’t always do it exactly how we had planned to do it. And I think I mentioned before that it just takes longer to do anything with the government. In some ways it’s sometimes easier to do something completely separate, so it takes longer and you have to have more patience, so I think that was a challenge.”*
	Local policies and management structure strength;	*“...it is not our decision, we do not decide where the facilities or nurses should be; it’s the community that decides because we also expect some responsibility from the community.”*	Public sector and other funding fluctuations	“*The … health system has tremendous logistics and stock out problems. From the very beginning of the study, we were required … to conduct integrated community case management of childhood illness, meaning that the CHWs had to provide antibiotics and antimalarials. There was no way through the health system that they could procure and deliver supplies to CHWs.”*
	Existing stakeholder capacity	*“…this is the program that also deals with a lot of NGOs. For instance, in our community health program the major stakeholder was…who have been already in the community and deal with many issues. So, in training community health nurses to work with the community, they were the people that we used.”*	Diseases prevalence	*“So, we recognize that the under 5 mortality was dropping but neonatal mortality wasn’t, it’s something that we recognize that a lot of the interventions were not actually addressing the needs of the babies.*”
			Human resources in the wider health System (attrition and shortage)	*“One of the challenges could be that there was a lot of staff movement. Not necessarily people leaving… So a health worker might move from one health center to another. So they would have been exposed to some of the activities but they are now in a different place. We had a couple key leadership people leave the project: the Chief Medical Officer at the provincial level, there was two of them throughout. So that’s a little challenge because you have to re-explain it.”*
Inner setting (academic and NGO implementing organizations)	Internal on the job mentorship and Capacity building	*“The goal behind that was that because this was a grant that was both implementation and research we really want to grow the capacity of people from…to actually participate in the research part of it but then I think recognizing the value of people being able to consume research and knowledge management and not just necessarily just produce research.”* *“I think expediently research capacity building beyond what was focused in the school of public health is something that was much broader. I think that was an adaptation, one [sic] was sort of really remarkable [sic] successful”*	Intra PHIT project Leadership Change; and Staff turnover requiring constant training Consistently training new staff due to turnover	*“I think changes in the statistical officers at the district level, that was a challenge because you have to keep going and re-training: it’s not linear, you can’t start something and keep gone [sic].”*
	Interdisciplinary team including National and International staff, and Expertise in local context	*“The institution I work for has been in existence since 2001, so certainly PHIT project was coming to fit into an institution that was already running. In fact, the institution had been doing a lot of health systems work before PHIT, but that was completely focused on ART, anti-retroviral therapy. In fact, my institution, I think it is fair to say that we shared data and championed the introduction of ART in...”*		
	Collaborative research approach	Increased research productivity and local and international collaboration. (Hedt-Gauthier, B. et al. 2017. BMC Health Services Research*, 2017.* Vol 17 Suppl 3. S3)		
	Multidisciplinary teams Strong content specific technical skills (PhD and implementation research track record; Academic partners)	“…*Strong Technical team. Statisticians and Epidemiologist as well as clinicians…”*		
Implementation process	Data use to inform iterations on intervention and implementation design	*“…the neonatal mortality reduction was definitely something we added on when we recognize [*sic*] that the under 5 mortality is [*sic*] dropping but neonatal mortality was not dropping.”*	Local intermittent change in data tools and methods.	*“It’s amorphous. Then it is in the system, the data systems for the ministry are constantly being updated and changing. So that’s a static thing. So, part of the project was to get, kind of started first making sure the data was okay, and try to figure out how to help them use it to make decisions, that would lead to management decisions is [*sic*] at the health facility but at the same time the data system will be updated and changed. So we have [*sic*] constantly kind of re-train people in their system, so it is not very linear.”*
	Cross intervention peer learning;	“*That year limiting was also a platform for us to know what is happening here, what is happening there, not only for the project itself in a hotel discussion but also field visits. I remember very well that the field visit was so exciting for us because we went there, we saw things that were happening, we learned, we pick lessons.”* *“We went to the field and we spent a whole day in the field to look at how the CHWs are working in the community. When we came back we had to talk about some of the findings, the challenges, the approach and that was changing the way because we were coming from a background with some experience. So for me, these interactions were not only for the benefit of each country but the entire PHIT program.”*		

## Results

### Intervention design and implementation

Four main themes emerged from the key informants’ descriptions of their experiences designing and implementing their HSS interventions. These included: 1) influence of context on intervention design; 2) intervention implementation and evolution; 3) PHIT cross-site interactions and influence on country-specific interventions, and; 4) perspectives for future HSS interventions.

#### Influence of context on intervention design

Context was reported to influence the design in several critical areas, including the structure and components of the intervention, as well as the original implementation strategy. These included the existing partnerships and new ones that needed to be established; relative strength of the existing health information system; capacity of the local and national health administration systems; and readiness of the partners in the targeted areas to implement. Interventions had similar approaches as well as differences depending on each of the country projects’ context.

Building on knowledge of key stakeholders and need to broaden engagement, PHIT project leaders each identified a number of shared core activities during the planning stage that were used to inform intervention design and implementation. These were focused on ensuring input and buy-in from key leaders and implementing partners (PHIT partnership members, national and local Ministry of Health leaders, local researchers, and district-level leaders) and sustaining and strengthening trust and acceptance from the community and intervention beneficiaries. This was accomplished through a participatory process for the design of the intervention that involved stakeholders from the local community level up to the Ministry of Health level, which was identified as critical to ensuring local priorities were addressed and trust was built with the community and partners. The public-private-academic partnership structure of each PHIT project team facilitated such diverse engagement. Other important preparatory work included securing a role for other existing development partners active in the area, ensuring readiness of the implementing teams to implement in specific areas, and integrating a monitoring and feedback system to inform needed responses to changing environment and other contexts for some (but not all) of the projects. This last approach differed from the fixed design of many previous interventions that had been implemented prior to the PHIT projects within each of the settings.

Reflecting fundamental strategies to improve population health and common contextual challenges, a number of core components were found to be shared in the intervention designs. All interventions involved two or more levels of the primary health system (e.g. community health workers, clinics, hospitals, district-or provincial-level management) recognizing that each level needed strengthening as well as coordination between the levels. Commonly employed intervention components to address these gaps included capacity building of health care workers including community health workers (CHWs); clinical and systems quality improvement, including mentoring, improved data quality and utilization; and strengthening of management and supervision systems [22–24]. However, some external contextual factors influenced the variability seen in some components. In Mozambique, the health system strengthening initiative was implemented in areas with high rates of health care service utilization at the baseline [14]. Therefore, the PHIT project opted to focus on improving data quality, data utilization, and management capacity to drive improvement in quality. In Ghana, expanding health care coverage was a priority, and the PHIT project leveraged an existing and well-funded CHW program (Community-based Health Planning and Services, or “CHPS”) to focus on interventions strengthening the community health service delivery model [13]. In Tanzania, the PHIT project reinforced the existing health system by introducing a new cadre of trained and compensated CHWs who provided diagnostic and therapeutic services to community members [15].

Faced with limited budgets and recognizing the need to design replicable interventions designed for sustainability, all PHIT project teams identified including an approach for leveraging and coordinating existing funds to support the intervention that reflected the local financial and management contexts as important to inform the development of successful interventions. Finally, PHIT team members described a strong commitment to building on existing collaboration with public sector-funded vertical programs and focusing on directly or indirectly strengthening the district health systems (as the lowest independent entity for primary health care system administration). This was mentioned as crucial to ensuring successful initiation of the interventions in all five countries. These components of the intervention were also seen as an integral step in planning for scale, integration, and sustainability from the onset of the intervention, although data on success in those areas was not available at the time of this study.

#### Intervention implementation and evolution

For projects to be successfully implemented, PHIT project leaders and their teams had to respond to different contextual realities that arose from the start and throughout the projects. For example, many of the new insights reflected challenges related to intervention components that had been designed based on assumptions made during the design phase by both the PHIT team and local partners. Each PHIT project rapidly adopted a flexible, iterative approach to best address those challenges. Project modifications included changing funding strategies, introducing new intervention components, adjusting project timelines, and extending interventions to different levels of the health system. For example, early in the implementation phase, one project leader recognized the need to more directly engage beneficiary communities, in addition to training CHWs, to maximize the success of the intervention, and adjusted their project accordingly.

*“Talking about learning, in the initial approach, we were trying to help the community without including them. What we learned from the Community Conversations process was that we need to work with the community in order to help them help themselves. The role of the community originally was less emphasized and originally it was much more around the CHWs that we are going to train, are coming from those communities so they know their communities well … But I think later we began to engage more with the entire community structure from the chief headman and structures within the community and challenge them to pick on activities that would address their health problems.”* – Key informant

Additionally, identified system-related weaknesses also informed adaptation. During start-up, one PHIT project recognized the need to create a supplementary supply chain to guarantee a steady and consistent supply of the equipment and drugs the CHWs needed to deliver the planned quality services.

*“The … health system has tremendous logistics and stock out problems. From the very beginning of the study, we were required … to conduct integrated community case management of childhood illness, meaning that the CHWs had to provide antibiotics and antimalarials. There was no way through the health system that they could procure and deliver supplies to CHWs…”* – Key informant

Another project found variation in health facility readiness across the facilities (e.g. infrastructure, staffing, and systems) that necessitated a rapid facility gap analysis and targeted investments to ensure that all facilities met basic standards. For several of the PHIT projects, having the flexibility to efficiently meet the constantly changing priorities and deadlines of the public system and local communities was noted as a challenge, but one which each PHIT project was able to accomplish.

*“Well, I just think the whole thing is kind of learning by doing, it’s like a cycle thing. That’s kind of embedded in our project, everything from the readiness of the health worker to understand their data, so in some cases we had to spend more time with them, in other cases we could spend less, but constantly adapting. We refined the presentations [of facility readiness and local performance]… as we went along, taking some indicators out, putting some indicators in-----we had to kind of refine how we help them frame the problems and the solutions because at first, the solution was something that was completely undoable like ‘build a new health center!’ … So all those little adaptations were constantly happening to those activities…”*– Key informant

Data utilization to identify challenges and inform these and other adaptations of the interventions was common. For example, results from baseline surveys that demonstrated high neonatal mortality led to the addition of neonatal care initiatives Ghana and Rwanda [25]. This approach resulted in health system improvements as well as alignment with the national government’s priorities by focusing on addressing key gaps within the existing health system. Additionally, Ghana, recognizing the challenges of emergency referral, added on a component of ambulances through a novel approach using converted motorbikes.

#### PHIT project cross-site interactions and influence on country-specific interventions

Creating a platform for knowledge exchange between PHIT projects was identified as an important contributing factor to each project’s decisions on how to adjust specific interventions to changing contextual factors in their setting through peer-learning and knowledge sharing. This exchange was achieved through the development of an overarching evaluation framework for the AHI [26] that created common metrics, annual AHI PHIT grantee meetings where projects shared both successes and challenges, and the creation of a Collaborative Management Committee that included representation from the leadership of each country project and was tasked with making recommendations to DDCF on other cross-site activities to strengthen the knowledge sharing [3, 26]. Each country hosted at least one of the AHI grantee meetings in the intervention areas during the project period, which provided the PHIT country team leaders from across AHI with the opportunity to witness the implementation of other projects first hand and talk with the frontline implementers and broad partnership stakeholders.

However, participants also mentioned the difficulty of adopting and implementing strategies that were successful in the other PHIT partnership projects within their own projects. Notable challenges included financial constraints for new intervention components within the fixed budget, introducing and getting acceptance from partners and stakeholders for new interventions or components during the active project, and limited capacity of the local public health system to take on additional project components.

#### Perspective for future HSS interventions

When considering the question, “If you were to implement a similar HSS intervention in the future, what would you change?” several respondents recommended a greater emphasis on understanding and achieving sustainability from the start. This emphasis should include identifying the ability of government or other partners to assume funding responsibility for successful innovations and a focus on strengthening local capacity to adopt best practices. All PHIT project teams identified that the end of the project period held many opportunities to respond to a range of health challenges through system-wide reforms. However, the projects also noted that the adoption and scale-up of successful health system strengthening intervention components by the public health sector were often limited by resource constraints, highlighting the critical need for effective evidence derived from integrated implementation research to prioritize high-value interventions.

*“If the ministry is going to allocate resources, priorities may be different: it may be equipment and rehabilitation of infrastructure and new infrastructure, and the service delivery component may be something which would be second tier in terms of funding. But if you are working in a resource-constrained environment, I think sustainability from a financial perspective, it’s a bit naïve to think about it that way.*” – Key informant

In some of the PHIT project countries, a number of intervention components are already in the process of sub-national or national scale up. In Ghana, planned national scale up of the strengthened CHPS program represents a 20-year commitment by the government and implementing partners to improve the CHPS model as a foundation of primary health service delivery [27]. In Rwanda, adaptation of the Mentoring and Enhanced Supervision for Health Care and Quality Improvement (MESH-QI) model was catalyzed by transition of PEPFAR funding to the Government of Rwanda, facilitating implementation of the mentorship approach for HIV care in up to 14 districts [28, 29]. In Tanzania, planning is underway for the training and deployment of Community Health Agents countrywide [15].

### Contextual factors influencing implementation success: (Table 2)

The analysis of the interview content using the CFIR to identify cross-site lessons highlighted the facilitators and challenges to implementation in the five chosen domains of the framework.

#### Intervention characteristics

Among the facilitators for implementation success, the innovations integrated into the intervention design were identified by all country projects as critical. One respondent reported that this was one of the first population health initiatives at the sub-national level in the country to take a comprehensive health systems approach focused on primary health care. The strategy to embed the intervention within the public health system, preventing paralleled systems and building on existing partnerships, also facilitated and accelerated implementation as local infrastructure and systems already existed. Specific components chosen to carefully reflect local needs and the quality of the design also helped the projects to go through local approvals faster, accelerating implementation. The commitment of many of the projects to utilize data to drive adaptation and embed a rigorous evaluation to inform this learning were also identified as important factors.

In the projects that did not integrate an ability to adapt into the study design, as was the case in Tanzania and Zambia, this approach proved to be challenging, especially when implementation faced contextual changes.

#### Outer setting

Local policies and management structure strength allowed more efficiencies in implementation and acceptance. The existence (and use) of these local management and implementation systems was an asset to PHIT projects. Reflecting the commitment to leverage these systems, the design facilitated a gap analysis and targeting of the intervention to address weaknesses in the existing system while leveraging existing strengths. In Ghana, Rwanda, Tanzania, and Zambia, the PHIT projects also leveraged support from the ministries of health and local public institutions to strengthen formal existing linkages between communities and district-wide system of health facilities. The Mozambique project intervened similarly but through the provincial health system level. The funding strategy of DDCF, encouraging a systems approach, which allowed for adaption and a relatively longer time frame of 5–7 years, was also identified as important.

In contrast, local policy change or evolution delayed or challenged the implementation. Changes like new administrations, national stock out, or public health system funding decline, informed substantial changes to PHIT interventions and sometimes delayed or canceled already implemented components of the interventions. Financial concerns have been raised by PHIT project teams when discussing sustainability of the PHIT projects.

#### Inner setting

PHIT projects shared a number of inner characteristics that influenced the implementation process. The track record of earlier collaborations with the local partners made the innovation of interventions and implementation more accepted locally. Examples included the internal mentorship and on the job capacity building in various domains of competencies and skills; the expertise that the local team had in the context; teamwork and collaboration on research initiatives to leverage all the skills available, local and international; and the culture of continuous learning and the ability to do so.

All of the projects shared that the readiness for implementation improved over time, often as a result of targeted investments in infrastructure and human resources (Rwanda), emergency transport systems (Ghana), and commodity supply chains (Tanzania). Even though the inner setting had multiple facilitators of the implementation process, challenges to those attributes were reported as not negligible. Human resource attrition and the cost of training and re-training health care workers; leadership change and resources to address the gap created by the changes, like constantly training CHWs, were commonly cited as major challenges.

#### Characteristics of the individuals involved

All PHIT projects reported that their teams were well received by the stakeholders and were recognized as adding new technical skills and expertise to the existing workforce. PHIT project teams had different levels of competencies and skills, which was reported to inspire confidence among the stakeholders.

All of the projects reported that knowledge transfer and capacity building were major priorities, with the only challenge being the sustainability of the newly trained cadre of community health workers in Zambia and Tanzania. For example, the Zambia, Rwanda, and Ghana projects initiated formal graduate-level research degree programs for local and national implementers in ministries of health and partner institutions.

#### Implementation process

Finally, informants described the implementation process as one of continuous learning.

All of the programs had to adjust their interventions to account for complex and dynamic contextual factors and re-design by introducing new innovations to improve the intervention or mend the new gaps developing over the course of implementation. For example, when baseline data in Rwanda demonstrated disproportionately high rates of neonatal deaths, the project expanded its quality improvement interventions to target neonatal survival. A common tension, particularly in Zambia and Tanzania, was determining whether, and how, to modify interventions, given that such changes could compromise the overall impact evaluations.

## Discussion

This paper has described the experience of implementing individual district- or provincial-level health system strengthening projects targeting primary health care in five sub-Saharan African countries: Ghana, Mozambique, Rwanda, Tanzania, and Zambia funded through a single initiative. Nearly 7 years after project initiation, our analysis identified five cross-cutting components that informed successful implementation and adaptation of contextually relevant HSS interventions. These included: ability to address health system challenges at multiple levels and sectors; ability to understand and incorporate local realities into intervention design; ability to accommodate the dynamics of real life changing context, with constant evaluation and integration of lessons throughout the intervention exercise; ability to learn across the PHIT projects and countries, to spread and adopt lessons learned; and the ability to keep sustainability at the core of design and in the approach to integrated lessons learned.

These lessons from the AHI model of funding come at a time when we are observing a substantial improvement in health indicators amidst sustained fundamental challenges to health systems in general. There has been reporting of reductions in premature mortality rates in recent decades, while an estimated 1 billion people still lack access to health care [30] and health systems, including primary health care, still fail to efficiently face challenges from current needs and emerging epidemics [31]. These insights from the AHI-funded projects which prioritized primary health care delivery are examples of how UHC can be achieved [31, 32]. The PHIT projects have shown that there is no one-size-fits-all approach to health systems strengthening. The diverse processes and nature of AHI interventions reflect the remaining, if not overlooked, gaps and priorities unique to each health system. Hence, academic, private, public, and community partnerships still need to be prioritized in order to leverage all available resources, and contextual knowledge and flexibility to adopt to changes are required.

In many ways, the AHI-funded interventions realized these overarching necessities. First, all projects intervened at multiple levels of the health system. Second, building on longstanding collaborations among the public, academic and private institutions that comprised the PHIT project, with local community partnerships, provided a foundation of confidence that facilitated efficient and effective implementation [5, 6]. Third, changes in policy and contextual factors required a dynamic implementation approach and evolution of interventions, though the latter often challenged the fidelity of impact evaluation. The ability to continuously evaluate the changes, together with the relatively long timeframe of the projects, provided time to integrate lessons learned during the implementation period. We think that this approach would allow projects in similar contexts to better address what were often chronic health system vulnerabilities [33, 34]. Fourth, improving institutional capacity required investments in the system and human capacity, ranging from management and governance to service delivery and research (e.g. in-service training, training at Masters and Ph.D level). We believe that these were core stepping stones for sustainability. Lastly, the final common component is the platform for learning and knowledge sharing across the AHI grantee network, which proved to be an important resource. This highlights the value of creating networks for knowledge transfer and spread of innovation in health systems research across the globe, and more importantly for growing health systems [3, 17, 26]. At a lower scale, the realization of specific deliverables was a key step toward the overall intended impact. Being able to continuously establish the relevance of those milestones allowed cross-site learning process and feasibility.

The intervention design and implementation approaches were reported to have facilitated sustainability and spread of components of projects. For example, the ongoing public sector scale-up of PHIT intervention components in Ghana, Tanzania, and Rwanda is encouraging, but also highlights the complexity of adapting and integrating research interventions into routine health system policy and implementation. The scale-up of the intervention in Ghana reflects a longstanding commitment of the government and its partners to the national strategy of CHPS, now informed by the results co-developed and shared between the public sector, academia, and implementing partners. In Rwanda, the expansion of the MESH-QI mentorship approach required substantial adaptation to take advantage of a partner-funded transition in the implementation of a vertical program, but was facilitated by the embedded research, which produced relevant evidence that was co-owned by government and implementing partners. In Tanzania, strong political will informed by lessons learned and shared from the intervention design and implementation results has led to the deployment of community health agents nationally [9, 27, 28, 35].

Despite the achievements, all PHIT partnership projects currently face the challenge of sustaining improvements and, in some cases, scaling up successful intervention components following completion of project interventions. Barriers to sustainability included weak health systems, workforce shortages and attrition, and lack of financial support [35]. However, the question of how to sustain long-term partnerships to continue to innovate and learn beyond the time period of a grant needs to be explored more in the future.

This analysis had a number of limitations. This analysis was not planned at the start of the projects, and the data collected was largely quantitative. We were also limited by time and resources and therefore reflected the insights from key members of the partnerships (ministry, academia, other local partners), but not the community or front-line implementers. We also could not conduct more than one or two interviews per project, which limited our ability to capture different viewpoints even within the PHIT partnership projects. Finally, any broader conclusions across the sub-Saharan African region will be limited by our sample size of five projects.

## Conclusion

The overall AHI grantee experience has shown that applying a systems thinking approach to health system strengthening interventions requires not only a comprehensive approach, but also a strong and efficient implementation framework. Complex partnerships with strong community, private, and public sector engagement, adaptation to local context, extended implementation timeframes to refine and improve interventions, using embedded research and committing to use data during implementation to adapt, as well as investments in institutional and human capacity, were key elements of successful and sustainable implementation. The question of how to sustain and scale successful HSS interventions targeting improvements at the primary health care level and how to effectively and efficiently integrate the capture of contextual factors and implementation process prospectively warrant further investigation.

## Abbreviations

AHI: African Health Initiative
CHPS: Community-based Health Planning and Services
CHW: community health worker CFIR Consolidated Framework for Implementation Research
DDCF: Doris Duke Charitable Foundation
HSS: health systems strengthening
MDG: Millennium Development Goal
MESH-QI: Mentoring and Enhanced Supervision for Health Care and Quality Improvement
NGO: non-governmental organization
PHIT: Population Health Implementation and Training
SDG: Sustainable Development Goal
UHC: Universal Health Coverage
UN: United Nations
WHO: World Health Organization

## References

[1] World Health Organization, 2009. System thinking for health system strengthening*.* Alliance f. A. Savigny, Don de, Taghreed, ed., Geneva: WHO.

[2] Somanje H (2009). Optimizing global health initiatives to strengthen national health systems. African Health Monitor.

[3] Sherr K (2013). Implementation research to catalyze advances in health systems strengthening in sub-Saharan Africa : the African health initiative. BMC Health Serv Res.

[4] Olmen, J. Van et al., 2012. The health system dynamics framework: the introduction of an analytical model for health system analysis and its application to two case-studies. Health Cult Soc, 2(1), pp.0–21.

[5] Bassett MT (2013). From the ground up: strengthening health systems at district level. BMC Health Serv Res.

[6] Drobac PC (2013). Comprehensive and integrated district health systems strengthening : the Rwanda population health implementation and training (PHIT) partnership. BMC Health Serv Res.

[7] Sachs, J.D., 2012. From Millennium Development Goals to Sustainable Development Goals - MDGs-to-SDGs. Lancet, p.202006.[online]. http://www.thelancet.com/pdfs/journals/lancet/PIIS0140-6736(12)60685-0.pdf. Accessed 2 Feb 2016. 10.1016/S0140-6736(12)60685-0 22682467

[8] Ottersen T (2017). Towards a coherent global framework for health financing: recommendations and recent developments. Health Econ Policy Law.

[9] Mutale W (2013). Systems thinking in practice : the current status of the six WHO building blocks for health system strengthening in three BHOMA intervention districts of Zambia : a baseline qualitative study. BMC Health Serv Res.

[10] Lewis A (2012). Lipstiz. Understanding health care as a complex system. The foundation of unintended consequences. JAMA.

[11] Tabak RG (2012). Bridging research and practice. Am J Prev Med.

[12] Peters DH (2014). Republished research : implementation research : what it is and how to do it implementation research is a growing but not. Br J Sports Med.

[13] Awoonor-Williams JK, Bawah A, Phillips JF (2013). The Ghana essential health interventions program: a plausibility trial of the impact of health systems strengthening on maternal & child survival. BMC Health Serv Res.

[14] Sherr K, Cuembelo F (2013). Strengthening integrated primary health care in Sofala, Mozambique. BMC Health Serv Res.

[15] Ramsey K (2013). The Tanzania connect project: a cluster-randomized trial of the child survival impact of adding paid community health workers to an existing facility- focused health system. BMC Health Serv Res.

[16] Stringer JSA (2013). Protocol-driven primary care and community linkages to improve population health in rural Zambia: the better health outcomes through mentoring and assessment (BHOMA) project. BMC Health Serv Res.

[17] Damschroder LJ (2009). Fostering implementation of health services research findings into practice: a consolidated framework for advancing implementation science. Implement Sci.

[18] Murphy AL (2014). A theory-informed approach to mental health care capacity building for pharmacists. Int J Ment Health Syst.

[19] Bunger AC, Hanson RF, Doogan NJ, Powell BJ, Cao Y, Dunn J (2016). Can learning collaboratives support implementation by rewiring professional networks?. Admin Pol Ment Health.

[20] Rosalind E (2017). Using the consolidated framework for implementation research (CFIR) to produce actionable findings: a rapid-cycle evaluation approach to improving implementation. Implement Sci.

[21] Sales AE (2016). Implementing goals of care conversations with veterans in VA long-term care setting: a mixed methods protocol. Implement Sci.

[22] Manzi A (2017). Mentorship and coaching to support strengthening healthcare systems: lessons learned across the five population health implementation and training partnership projects in sub-Saharan Africa. BMC Health Serv Res.

[23] Sherr K (2017). Measuring health systems strength and its impact: experiences from the African health initiative. BMC Health Serv Res.

[24] Wagenaar B (2017). Data-driven quality improvement in low-and middle-income country health systems: lessons from seven years of implementation experience across Mozambique, Rwanda, and Zambia. BMC Health Serv Res.

[25] Magge H (2017). Tackling the hard problems: implementation experience and lessons learned in newborn health from the African health initiative. BMC Health Serv Res.

[26] Bryce J (2013). A common evaluation framework for the African health initiative. BMC Health Serv Res.

[27] Awoonor JK (2015). Catalyzing the scale-up of community-based primary healthcare in a rural impoverished region of northern Ghana. Int J Health Plann Mgmt.

[28] Manzi A, Magge H, Redditt V (2013). Nurse mentorship to improve the quality of health care delivery in rural Rwanda. Nurs Outlook.

[29] Rwanda Biomedical Center, 2013. Rwanda HIV and AIDS National Strategic Plan: July 2013 - June 2018. Kigali: Government of Rwanda-Ministry of Health.

[30] Tangcharoensathien V (2015). Accelerating health equity: the key role of universal health coverage in the sustainable development goals. BMC Med.

[31] Canceda C (2016). Strengthening health systems while responding to a health crisis lessons learned by a nongovernmental organization during the Ebola virus disease epidemic in Sierra Leone. J Infect Dis.

[32] Chapman J (2015). Emotionally durable design: objects.

[33] Farmer, P., 2013. Clinical trials and global health equity. The Lancet Global [online]. http://globalhealth.thelancet.com/2013/07/08/clinical-trials-and-global-health-equity, accessed February 5, 2016.

[34] Loewenson R (2014). Participatory action research in health systems: a methods reader, TARSC, AHPSR, WHO, IDRC Canada, EQUINET, Harare.

[35] Iwelunmor J (2016). Toward the sustainability of health interventions implemented in sub-Saharan Africa: a systematic review and conceptual framework. BMC Implement Sci.

